# Lower miR-21/ROS/HNE levels associate with lower glycemia after habit-intervention: DIAPASON study 1-year later

**DOI:** 10.1186/s12933-022-01465-0

**Published:** 2022-03-04

**Authors:** Lucia La Sala, Elena Tagliabue, Simona Mrakic-Sposta, Anna Chiara Uccellatore, Pamela Senesi, Ileana Terruzzi, Emilio Trabucchi, Luigi Rossi-Bernardi, Livio Luzi

**Affiliations:** 1 IRCCS, MultiMedica, PST-Via Fantoli 16/15, 20138 Milan, MI Italy; 2 Institute of Clinical Physiology, National Research Council (CNR), 20162 Milan, Italy; 3 ICCS Istituto Clinico Città Studi, Milan, Italy; 4 Dept. of Biomedical Sciences for Health, University of Milan, Milan, Italy; 5 Milan, Italy

**Keywords:** Dysglycemia, Prediabetes, microRNA-21, miR-21, ROS, Hydroxynonenal, Habit-intervention, Mediterranean diet, Glucose monitoring

## Abstract

**Background:**

The prevalence of prediabetes is increasing in the global population and its metabolic derangements may expose to a higher risk to develop type 2 diabetes (T2D) and its cardiovascular burden. Lifestyle modifications might have considerable benefits on ameliorating metabolic status. Alternative biomarkers, such as circulating miR-21, has been recently discovered associated with dysglycemia. Here we evaluated, in a longitudinal cohort of dysglycemic population the relation between the circulating miR-21/ROS/HNE levels and the habit-intervention (HI) after 1 year of follow-up.

**Methods:**

1506 subjects from DIAPASON study were screened based on the Findrisc score. Of them, 531 subjects with Findrisc ≥ 9 were selected for dysglycemia (ADA criteria) and tested for circulating miR-21, ROS and HNE levels, as damaging-axis. 207 subjects with dysglycemia were re-evaluated after 1-year of habit intervention (HI). Repeated measures tests were used to evaluate changes from baseline to 1-year of follow-up. The associations between glycemic parameters and miR-21/ROS/HNE were implemented by linear regression and logistic regression models.

**Results:**

After HI, we observed a significant reduction of miR-21/ROS/HNE axis in dysglycemic subjects, concomitantly with ameliorating of metabolic parameters, including insulin resistance, BMI, microalbuminuria, reactive hyperemia index and skin fluorescence. Significant positive interaction was observed between miR-21 axis with glycaemic parameters after HI. Lower miR-21 levels after HI, strongly associated with a reduction of glycemic damaging-axis, in particular, within-subjects with values of 2hPG < 200 mg/dL.

**Conclusions:**

Our findings demonstrated that HI influenced the epigenetic changes related to miR-21 axis, and sustain the concept of reversibility from dysglycemia. These data support the usefulness of novel biological approaches for monitoring glycemia as well as provide a screening tool for preventive programmes.

**Supplementary Information:**

The online version contains supplementary material available at 10.1186/s12933-022-01465-0.

## Introduction

Dysglycemia or prediabetes is associated with cardiovascular risk [1] and is mostly described for cardiovascular mortality [2]. Dysglycemia, belonging to the spectrum of glucose abnormalities, arises silently and years before the clinical manifestation [3]; it includes isolated impaired fasting glucose (IFG) and/or isolated impaired glucose tolerance (IGT), exposing people to the risk of type 2 diabetes (T2D) development. Glucose intolerance is detected using a 75-g glucose load test where IGT is defined as a 2-h plasma glucose (2hPG) value of 140–199 mg/dL (7.7–11.1 mmol/L), or IFG of 100–125 mg/dL, or HbA1c of 5.7–6.4% (39–46 mmol/mol) [4, 5]. Recently, the measurements of 1hPG are emerging to define the prediabetic and diabetic status [6, 7] although the consensus on the diagnostic definition of pre-diabetes is still debated [8].

Prediabetes is considered a target for lifestyle intervention in virtue of its peculiarity of “multifactorial and heterogeneous condition” characterized by an asymptomatic transitional state of hyperglycaemia. Therefore, prediabetes—sharing with T2D insulin resistance (IR), over-weight and obesity, which are modifiable risk factors for other cardiometabolic risk factors, such as hypertension [9]—is more liable to a higher risk of the development of cardiovascular disorders [10].

In light of this scenario, preventive programs are focusing on the modification of lifestyle, that notoriously decreases the incidence of T2D, to reduce the risk and delay T2D occurrence and its burden: Diabetes Prevention Program (DPP) recommended healthy habits (diet and physical activity), avoiding smoking, alcohol, and stress as hints to reduce the risk to develop T2D. Intensive lifestyle intervention was able to reduce the incidence of T2D by 58% over 3 years [11, 12].

In the last decade, alternative preventive strategies have focused on genetic approaches, through the investigations on SPNs, to identify loci susceptibility in assessing the risk to develop T2D. However, a strict interaction between unhealthy diet and genetic predisposition was established in cancer [13], but there are inconclusive results about the interactions among common genetic variants, Western diet and lifestyle risk factors on the risk to develop T2D [14]. On the other hand, environmental stimuli, nutritional factors, inflammation, hypoxia, arterial pressure, sex, age, and lifestyle can force changes in epigenetic factors, explaining the heterogeneous phenotypes related to complex and multifactorial diseases, such as dysglycemia. It has been known that dysregulation of epigenetic processes has important consequences for the pathogenesis of T2D and vascular complications [15, 16]. micro-RNAs (miRNAs or miRs), belonging to the epigenetic cluster, may contribute to ascertaining the main mechanisms for dysglycemia and provide a novel tool for a precision preventive strategy. miRNAs, known as highly conserved non-coding RNAs of 25 nucleotides in length, are important forms of biological regulation involving the control of gene expression that may be potentially reversible without altering the DNA sequence [17] because is a process that occurs on the post-transcriptional level. In virtue of their association with the extracellular vesicles (EVs), or carrier proteins, miRNAs may be up-taken by recipient cells influencing their functions [18], and confer high stability in the circulation, making them as biomarkers for T2D, for insulin metabolism, glucose homeostasis [19], and diabetes complications [20, 21]. Current evidence has demonstrated that miRs may predict the progression from pre-diabetic to diabetes state [22]. Many studies reported that glucose, as a nutrient, was able to influence cellular oxidative milieu [23] and miRs expression [24]. Indeed, a particular diet may change the responsiveness of several endogenous miRs in animals [25, 26].

Our previous works demonstrated the ability of the glucose-sensing miR-21 to predict ROS (reactive oxygen species)-mediated damage in prediabetic and T2D subjects, suggesting a role of miR-21 as a novel approach for monitoring glucose, and their relative damage [27, 28].

In this study, we sought to evaluate whether miR-21 damaging-axis would be downregulated by habit intervention (HI, mostly composed by Mediterranean Diet) after one year of follow-up (in high-risk subjects that at baseline showed dysglycemia and a ROS induced damage) delineating with a good approximation a novel mechanism for monitoring damage glycemia-induced.

## Research design and methods

### Participants and setting

The longitudinal cohort of the DIAPASON (Diabetes Prediction And Screening Observational) Study—a diabetes prevention programme—was used for estimating prediabetes by a procedure based on the Finnish Diabetes Risk Score (FINDRISC) [29]. 1506 participants were selected based on eligible criteria by general practitioners (GP) of Milan and invited for signed informed consent before a laboratory screening. Age of 40–75 years and Findrisc score ≥ 9 were the eligibility criteria adopted [30]. Dysglycemia condition was defined by American Diabetes Association (ADA) as at least one of the glycemic parameters, FPG, 2hPG and HbA1C (respectively, > of 5.6 mmo/L (100 mg/dL), 7.8 mmol/L (140 mg/dL) and 5.7% (39 mmol/mol) [5]). Skin intrinsic fluorescence (SIF) was measured as autofluorescence in human skin with the use of an AGE-Reader (DiagnOptics Technologies BV), that can estimate the accumulation of AGE (advanced glycated end products) in the skin. SIF was determined from the ratio between the emission and reflected excitation light (ratio between the light intensity reflected in the 420–600 nm wavelength range and the light intensity in the 300–420 wavelength) using the AGE-Reader software. EndoPAT 2000 (Itamar Medical, Israel) evaluates acute changes in endothelial function, expressed as RHI (reactive hyperemia index). Subjects were in the supine position for 20 min before measurements, in a quiet, temperature-controlled room with dimmed lights. Each recording consisted of 5 min of baseline/5 min of occlusion/5 min post-occlusion measurement (hyperemic period). Occlusion of the brachial artery was performed on the non-dominant upper arm. The occlusion pressure was at least 60 mmHg above the systolic blood pressure (minimally 200 mmHg, and maximally 300 mmHg).

The DIAPASON protocol was approved by the institutional review boards of the IRCCS MultiMedica [protocol number 24/2012(153)]. Participants were recruited between January 2013 and February 2017. All subjects gave written informed consent.

### Selection criteria for habit-intervention (HI) protocol

According to the FINDRISC questionnaire, at baseline, all subjects were at high risk of developing T2D. 207 subjects that at baseline showed at least one glycemic diagnostic (preferentially based on HbA1c) parameter altered was encouraged to correct lifestyle.

A diet was assigned according to the Mediterranean diet (MedDiet) scheme (50–55% carbohydrates, 15–20% proteins, 25–30% lipids) with the caloric quantity chosen for each subject based on the basal metabolic rate calculated with Harris & Benedict formulas [31].

### Selection criteria for miR-21 responder group characterization

We selected as Responders group (R) all subjects that, after HI, showed lower miR-21 plasmatic levels than baseline. Subjects (16%) with no changes in miR-21 levels after HI were classified as the Non-Responders group (NR).

### Plasma separation and laboratory testing

Peripheral blood sample (5 mL) was extracted in a tube with ethylenediaminetetraacetic acid (EDTA) as an anticoagulant, at room temperature, and centrifuged at 3000 r/min for 10 min. Serum and plasma were separated as routine assessments. Fasting plasma glucose (FPG) was detected by the Slein method using Siemens analyser (Germany); oral glucose tolerance test (OGTT, 75 gr glucose in 300 mL) was used to assess the 2hPG and 1hPG values. Triacylglycerol and total cholesterol were measured using an automated enzymatic colourimetric test (Siemens). HbA1c was detected by a high-performance liquid chromatography automated system (Tosoh, Japan). Insulinemia levels were detected by a Centaur XP analyzer (Siemens). HOMA-IR was calculated by the formula ‘FPG (mg/dL) × fasting insulin (uU/mL)/405’. m-ALB was detected in urine samples previously centrifuged for 10 min at 3000×*g* to avoid cellular debris using an IMMAGE instrument (Beckman Coulter).

### RNA extraction and miRNA determination with real-time PCR analysis

100 μL of plasma was processed for total RNA extraction using an RNA purification kit (NorgenBiotek, Thorold, ON, Canada) following the manufacturer’s instructions. Plasma was centrifuged at 13,000*g* for 5 min at 4 °C to avoid platelets interferences. Before RNA extraction, 5 μL of *cel*-miR-39 [(synthetic *Caenorhabditis elegans*-miR-39), purchased by Applied Biosystems, Life Technologies, Grand Island, NY, USA] was spiked into the plasma to ensure efficiency RNA recovery. The TaqMan MicroRNA Reverse Transcription Kit (Thermo-Fisher) was used to reverse-transcribe miRNAs as recommended by the manufacturer. Real-time qPCR for plasmatic measurements of miR-21 levels was performed with a QuantStudio 6 flex (Applied Biosystems, Foster City, CA, USA) detection system. Data were obtained as Ct values, and the 2 − ΔCt method was applied for analysis of miRNA expression levels, normalizing with external cel-miR-39. In addition, to assess the hemolysis of the samples the ratio between absorbance at 414 nm and 375 nm was calculated [32].

### Determination of ROS by electron paramagnetic resonance (EPR)

ROS generation was detected in sera by X band-EPR spectroscopy (E-scan—Bruker BioSpin, GmbH, MA USA) using the EPR method [33, 34]. Sera were incubated with 1 mM CMH (1-hydroxy-3-methoxycarbonyl-2,2,5,5-tetramethylpyrroline) probe prepared in buffer (Krebs-Hepes buffer (KHB) containing 25 μM deferoxamine methane-sulfonate salt (DF) chelating agent and 5 μM sodium diethyldiothio-carbamate trihydrate (DETC) at pH 7.4). An external reference, stable radical CP· (3-Carboxy2,2,5,5-tetramethyl-1-pyrrolidinyloxy) was used to convert ROS determinations in absolute quantitative values (μmol.min^−1^). All spectra recorded were analysed by standard software (Win EPR 2.11, Bruker).

### Determinations of plasmatic 4-hydroxynonenal (HNE) and asymmetrical dimethylarginine (ADMA)

One hundred μL of plasma samples in duplicate were used to analyse HNE-protein adduct concentrations with a commercially available immunoassay kit following the manufacturer’s instructions (Oxiselect, Cell Labs, San Diego, CA, USA). One hundred μL of plasma samples in duplicate were used to analyse asymmetrical dimethylarginine (ADMA) concentration with a commercially available immunoassay kit following the manufacturer’s instructions (Cusabio, Houston, TX, USA).

### Statistical analysis

Data were presented as mean ± standard deviation and compared between baseline and after 1 year of follow-up by using the Wilcoxon Signed-Rank test or paired T-test, as appropriate. Comparisons between sex were analysed by T-test or non-parametric Wilcoxon test, as appropriate. The correlation matrix for miR-21/ROS/HNE and other collected variables at follow-up was evaluated. Logistic regression models to evaluate the association between R group and each glycaemic parameter were performed and receiver operating characteristic (ROC) curves were drawn. For each model odds ratio (OR) with 95% CI (confidence interval) and area under the curve (AUC) were calculated. To evaluate the association between ROS/miR-21 values and glycaemic parameters, linear regression models were implemented. All statistical analyses were performed with SAS Software 9.4 and graphs were drawn by GraphPrism v7. P-values < 0.05 were considered statistically significant for all analyses.

## Results

### Healthy habit intervention (HI) positively influences cardiometabolic traits

Participants at baseline (N = 531) with a moderate/high risk to develop T2D, calculated by Findrisc score ≥ 9, and with at least one of glycemic diagnostic criteria impaired, were encouraged to achieve weight loss and reduce waist circumference, known risk factors for cardiometabolic diseases, recommending a combination of the Mediterranean diet with behavioural support. At 1 year of follow-up, N = 207 subjects were re-evaluated.

### Characteristics of subjects

At baseline the cohort had a mean age of 59.7 (± 8.8) years, 57.4% were women. 37.5% of the participants were at slightly elevated risk (FINDRISC 9–11).

The analysis for repeated measures (Table 1) showed significant changes between baseline and after HI 1 year later: overall, BMI was reduced (p = 0.004) and the trend was more evident in women than in men; waist circumference was reduced (p < 0.0001) for both women and men significantly. Also, we notice SIF (p = 0.005) and HbA1C reduction (p < 0.0001), although other glycemic parameters showed a tendency to reduce their values but unreached significance. The increase of HDL is significant, whereas insulinemia, HOMA-IR and micro-albuminuria (m-ALB) were reduced (p < 0.0001, p < 0.0001 and p = 0.04, respectively). Regarding endothelial function measured by EndoPAT, reactive hyperemic index (RHI) showed reduced values (p = 0.015). Augmentation index normalized to 75 bpm (%) as the index of arterial stiffness, showed a tendency to decrease but unreached significance, together with the reduction of heart rate variability (HRV) and asymmetrical dimethylarginine (ADMA), an endogenous competitive inhibitor of nitric oxide (NO) synthesis, used for assessment of endothelial dysfunction (ED) [35].

**Table 1:** Characteristics of participants

	Baseline	HI 1-yr	N paired	p-value
Age	59.7 ± 8.8			
Women (%)	57.4	55.6		
DBP (mm Hg)	76.2 ± 11.5	76.5 ± 11.4	204	0.6627
SBP (mm Hg)	128.8 ± 15.4	129.4 ± 14.2	204	0.6412
Cardiac frequency (bpm)	70.3 ± 8.1	70.6 ± 8.2	201	0.9933
BMI (Kg/m^2^)	27.5 ± 4.3	27.2 ± 4.2	203	**0.0040**
Woman	27.3 ± 4.9	26.9 ± 4.9	113	**0.0177**
Man	27.8 ± 3.4	27.5 ± 3.2	90	0.0928
Waist circumference (cm)	97.6 ± 10.2	95.3 ± 10.2	195	** < .0001**
Woman	95.0 ± 10.4	93.3 ± 10.9	108	**0.0127**
Man	100.9 ± 9.1	97.7 ± 8.8	87	** < .0001**
RHI (a.u.)	2.41 ± 0.76	2.27 ± 0.68	189	0.0152*
AI (75 bpm%)	22.1 ± 19.9	21.7 ± 19.8	191	0.6636
HRV (a.u.)	39.3 ± 36.2	35.1 ± 17.6	188	0.5246
SIF (fluor. unit)	2.26 ± 0.47	2.15 ± 0.46	196	**0.0059**
FPG (mg/dL)	95.1 ± 14.0	94.5 ± 15.7	204	0.3941
1hPG (mg/dL)	170.9 ± 45.6	169.7 ± 61.1	196	0.7554*
2hPG (mg/dL)	141.4 ± 49.8	137.6 ± 48.9	201	0.1334*
HbA1C (%)	6.2 ± 0.4	6.0 ± 0.4	201	** < .0001**
HbA1C (mmol)	44.7 ± 4.6	42.1 ± 4.2	204	** < .0001**
Col (mg/dL)	207.7 ± 37.4	206.5 ± 36.7	204	0.9169
HDL (mg/dL)	56.0 ± 14.9	58.6 ± 16.2	207	** < .0001**
TAG (mg/dL)	120.6 ± 65.0	116.7 ± 53	207	0.8022
LDL (mg/dL)	127.8 ± 32.5	124.8 ± 31.2	207	0.2645
INS (mIU/L)	19.5 ± 26.4	13.5 ± 11.6	200	** < .0001**
HOMA-IR	4.7 ± 6.2	3.3 ± 3.4	195	** < .0001**
m-ALB (mg/dL)	15.0 ± 34.5	11.7 ± 16.2	184	**0.0385**
mir-21 (a.u)	0.035 ± 0.039	0.011 ± 0.022	116	** < .0001**
HNE (ug/mL)	9.1 ± 9.2	6.5 ± 5.8	130	** < .0001**
ADMA (ng/mL)	51.7 ± 50.9	45.5 ± 24.5	85	0.3391
ROS (umol/min)	0.204 ± 0.017	0.195 ± 0.020	48	0.0285*

N = 207 subjects of the N = 531 enrolled in the DIAPASON study were re-evaluated after 1 year of follow-up under healthy-habit intervention

miR-21: circulating microRNA-21; HI 1-yr: habit intervention of 1 year; DBP: diastolic blood pressure; SBP: systolic blood pressure; BMI: body mass index; RHI: reactive hyperemia index; AI: augmentation index; HRV: heart rate variability; SIF: skin intrinsic fluorescence; FPG: fasting plasma glucose; 1hPG: 1-h plasma glucose; 2hPG: 2-h plasma glucose; HbA1C: glycated hemoglobin; Col: total cholesterol; HDL: high-density lipoprotein; TAG: triacylglycerol; LDL: low-density lipoprotein; INS: insulinemia; HOMA-IR: homeostatic model assessment for insulin resistance; m-ALB: microalbuminuria; HNE: hydroxynonenal; ADMA: asymmetrical dimethylarginine; ROS: reactive oxygen species

Wilcoxon Signed-Rank test; * Paired T-test

### Effect of habit-changes on circulating miR-21/HNE/ROS axis 1 year later

**Fig. 1:**
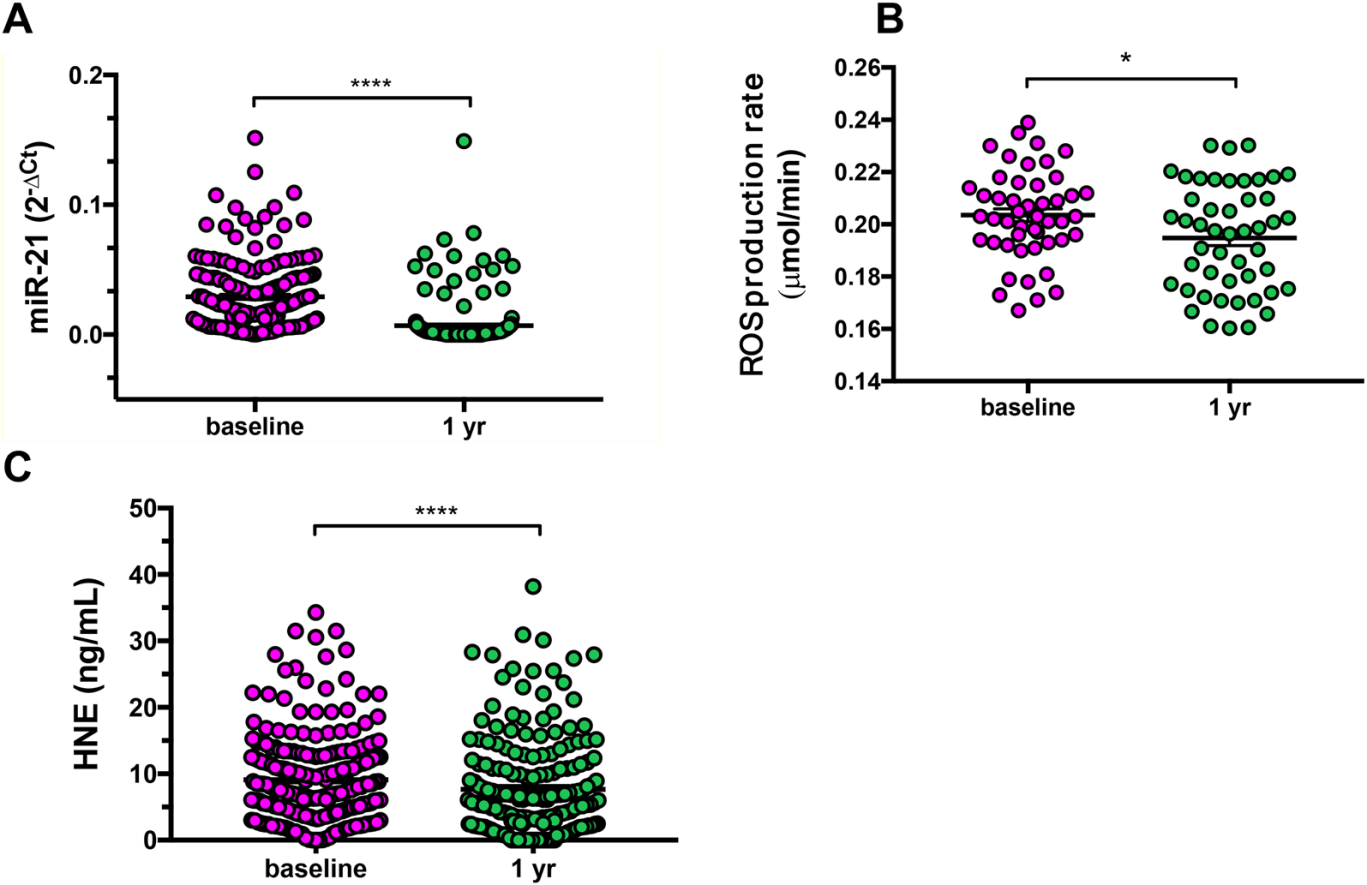
miR-21 expression in plasma samples after 1 year of habit-intervention (HI). **A** Scatter dot plot of miR-21 expression at baseline and 1 year after habit-intervention in which miR-21 was normalised to cel-miR-39 expression using the comparative Ct method in a longitudinal cohort (at baseline, N = 531; at 1 year of HI, n = 207; paired miR21, n = 116). Wilcoxon Signed-Rank test for paired data, p < 0.0001. **B** Scatter dot plot of ROS extracellular release (measured by EPR instruments) in sera showing significant reduction after habit-intervention (ROS paired n = 48). Paired T-test, p = 0.03. **C** Scatter dot plot of plasma concentration of HNE (μg/mL) in the dysglycemic cohort (paired HNE n = 130). Wilcoxon Signed-Rank test for paired data, p < 0.0001. ****p < 0.0001; *p < 0.05.

The subjects with higher risk at baseline, determined by Findrisc score, and with at least one of the glycemic criteria impaired, were re-evaluated 1 year after HI, in which subjects become aware of their risk and decided to change their habits ameliorating nutrition and avoid sedentarily. At baseline we found in dysglycemic subjects, increased miR-21/ROS/HNE levels, as reported in our previous work [28], suggesting regulation underlying epigenetic mechanisms. qPCR was used for miR-21 detection in the plasma of subjects (N = 116). Our data showed that the mean value of miR-21 plasma level was decreased after 1 year of HI (p < 0.0001) (Table 1; Fig. 1A), suggesting a strict relationship between miR-21 and glycaemic statuses.

#### Lifestyle changes reduce both oxidative stress response

Serum was used to detect ROS absolute concentration values by EPR (Fig. 1B), although it clouded the main site of ROS production. After 1-year of HI, the dysglycemic subjects had reduced ROS levels with significant differences compared to baseline (p = 0.03) (Table 1; Fig. 1B). To investigate the free radical-induced lipid damage occurring in plasma and the relative effectiveness of HI power in preventing this damage, we assessed the presence of specific end products of peroxidized lipid as HNE-protein adducts, at baseline and after HI 1 year later. However, the levels were significantly lower when we compared baseline and HI, unveiling a status of improved ROS/HNE-damage in dysglycemia pathogenesis (Table 1; Fig. 1C).

Interestingly, after adjustments of lifestyle, also the correlation analysis (Additional file 1: Table S1) revealed a significant positive association of miR-21 with glycaemic parameters, such as FPG, 1hPG and 2hPG, HbA1C (% and mmol/mol), HNE (*rho* = 0.2, p = 0.03) and ROS generation (*rho* = 0.5, p = 0.0002). ADMA showed lower levels after HI, but did not reach significance.

#### Effect of habit-changes on Responders group

Dysglycemic subjects who after HI significantly reduced miR-21 (classified as Responders, or R, Additional file 1: Table S1) exhibited the greatest metabolic changes (Table 2), compared with those miR-21 unchanged (Non-Responders, or NR) (Table 3). Intriguingly, in R group we found similar miR-21-values in cut-off for discriminating dysglycemic subjects as evaluated in our previous published results [28].

**Table 2:** Characterization of the subjects with lower-miR-21 (Responders)

	Baseline	HI 1-yr	N paired	p-value
DBP (mm Hg)	76.5 ± 12.6	77.1 ± 11.7	97	0.6729
SBP (mm Hg)	129.8 ± 16.8	130.4 ± 14.1	97	0.7637*
Cardiac frequency (bpm)	70.5 ± 8.0	70.8 ± 8.6	95	0.7702*
BMI (Kg/m^2^)	27.5 ± 4.4	27.0 ± 4.0	96	**0.0036**
Waist circumference (cm)	97.2 ± 10.2	95.0 ± 9.4	91	0.0015*
RHI (a.u.)	2.415 ± 0.756	2.197 ± 0.607	92	**0.0275**
AI (75 bpm%)	22.0 ± 19.6	20.2 ± 19.2	91	0.4471
HRV (a.u.)	43.1 ± 47.3	35.2 ± 19.3	90	0.1595
SIF (fluor. unit)	2.255 ± 0.393	2.206 ± 0.399	95	0.4586
FPG (mg/dL)	96.1 ± 12.3	94.0 ± 10.4	97	0.1924
1hPG (mg/dL)	178.9 ± 41.8	174.7 ± 69.1	94	**0.0080**
2hPG (mg/dL)	149.4 ± 42.6	138.3 ± 38.7	96	0.0540*
HbA1C (%)	6.2 ± 0.4	6.0 ± 0.4	95	** < .0001**
HbA1C (mmol)	44.6 ± 4.0	42.2 ± 4.1	97	** < .0001**
Col (mg/dL)	206.1 ± 34.6	204.1 ± 37.1	97	0.5061*
HDL (mg/dL)	54.0 ± 13.4	56.4 ± 15.2	97	**0.0081**
TAG (mg/dL)	123.3 ± 61.3	118.9 ± 47.5	97	0.9806
LDL (mg/dL)	127.4 ± 30.5	123.9 ± 32.3	97	0.2220*
INS (mIU/L)	18.3 ± 21.1	14.1 ± 12.5	97	**0.0007**
HOMA-IR	4.4 ± 4.9	3.4 ± 3.3	96	**0.0004**
m-ALB (mg/dL)	14.9 ± 21.1	12.1 ± 15.7	83	**0.0230**
HNE (ug/mL)	8.2 ± 7.7	5.3 ± 4.1	95	** < .0001**
ADMA (ng/mL)	47.5 ± 45.9	43.8 ± 22.2	62	0.1196
ROS (umol/min)	0.204 ± 0.017	0.19 ± 0.018	33	0.0045*

Women				
DBP (mm Hg)	72.8 ± 11.4	75.4 ± 11.5	55	0.1999
SBP (mm Hg)	126.8 ± 17.0	130.7 ± 14.6	55	0.1471*
Cardiac frequency (bpm)	72.4 ± 7.9	72.6 ± 9.3	53	0.9135*
BMI (Kg/m^2^)	27.3 ± 4.9	26.7 ± 4.6	55	0.0337*
Waist circumference (cm)	94.8 ± 10.1	93.1 ± 9.7	52	0.1170*
RHI (a.u.)	2.555 ± 0.843	2.256 ± 0.649	52	**0.0328**
AI (75 bpm%)	27.3 ± 19.3	24.2 ± 18.1	52	0.1943*
HRV (a.u.)	34.4 ± 20.1	33.3 ± 19.3	51	0.3973
SIF (fluor. unit)	2.296 ± 0.417	2.246 ± 0.369	54	0.9844
FPG (mg/dL)	93.4 ± 12.3	92.0 ± 10.4	55	0.6405
1hPG (mg/dL)	178.0 ± 45.4	178.3 ± 88.4	52	**0.0296**
2hPG (mg/dL)	145.5 ± 47.5	138.7 ± 41.8	54	0.1536*
HbA1C (%)	6.3 ± 0.3	6.1 ± 0.4	54	** < .0001**
HbA1C (mmol)	45.2 ± 3.6	42.7 ± 3.9	55	** < .0001**
Col (mg/dL)	211.7 ± 35.7	211.1 ± 34.1	55	0.8807*
HDL (mg/dL)	59.0 ± 13.1	62.3 ± 15.0	55	**0.0058**
TAG (mg/dL)	111.9 ± 47.8	113.9 ± 43.0	55	0.2337
LDL (mg/dL)	130.4 ± 29.9	126.0 ± 29.8	55	0.2092*
INS (mIU/L)	16.8 ± 18.8	11.9 ± 8.7	55	**0.0002**
HOMA-IR	3.9 ± 4.0	2.7 ± 1.9	55	**0.0002**
m-ALB (mg/dL)	14.3 ± 22.5	10.5 ± 11.7	46	0.0955
HNE (ug/mL)	8.5 ± 6.6	6.0 ± 4.3	54	**0.0001**
ADMA (ng/mL)	45.5 ± 39.9	43.1 ± 24.1	32	0.1642
ROS (umol/min)	0.203 ± 0.021	0.19 ± 0.019	15	0.1366*

Men				
DBP (mm Hg)	81.4 ± 12.5	79.4 ± 11.6	42	0.4353*
SBP (mm Hg)	133.7 ± 15.9	130.0 ± 13.7	42	0.1945
Cardiac frequency (bpm)	68.0 ± 7.4	68.5 ± 7.3	42	0.7313*
BMI (Kg/m^2^)	27.9 ± 3.6	27.5 ± 3.2	41	0.0691
Waist circumference (cm)	100.6 ± 9.5	97.5 ± 8.5	39	0.0006*
RHI (a.u.)	2.233 ± 0.586	2.121 ± 0.547	40	0.3240*
AI (75 bpm%)	14.8 ± 17.9	14.8 ± 19.4	39	0.9883*
HRV (a.u.)	54.4 ± 67.0	37.8 ± 19.2	39	0.1983
SIF (fluor. unit)	2.20 ± 0.356	2.15 ± 0.435	41	0.2293
FPG (mg/dL)	99.7 ± 11.3	96.7 ± 9.8	42	0.1801
1hPG (mg/dL)	180.1 ± 37.4	170.2 ± 32.6	42	0.0713*
2hPG (mg/dL)	154.5 ± 35.2	137.8 ± 34.7	42	0.0145*
HbA1C (%)	6.2 ± 0.4	6.0 ± 0.4	41	** < .0001**
HbA1C (mmol)	43.9 ± 4.5	41.6 ± 4.2	42	** < .0001**
Col (mg/dL)	198.9 ± 32.2	194.9 ± 39.3	42	0.4210*
HDL (mg/dL)	47.6 ± 10.9	48.6 ± 11.4	42	0.3664
TAG (mg/dL)	138.2 ± 73.4	125.4 ± 52.7	42	0.3812
LDL (mg/dL)	123.6 ± 31.3	121.3 ± 35.4	42	0.6266*
INS (mIU/L)	20.2 ± 23.8	17.1 ± 15.9	42	0.2601
HOMA-IR	5.1 ± 5.9	4.2 ± 4.4	41	0.1684
m-ALB (mg/dL)	15.6 ± 19.5	14.0 ± 19.5	37	0.0991
HNE (ug/mL)	7.8 ± 9.0	4.4 ± 3.5	41	** < .0001**
ADMA (ng/mL)	49.6 ± 52.0	44.6 ± 20.5	30	0.3911
ROS (umol/min)	0.204 ± 0.014	0.189 ± 0.018	18	0.0126*

Subjects after HI were characterized for lower circulating levels of miR-21

miR-21: circulating microRNA-21; HI 1-yr: habit intervention of 1 year; DBP: diastolic blood pressure; SBP: systolic blood pressure; BMI: body mass index; RHI: reactive hyperemia index; AI: augmentation index; HRV: heart rate variability; SIF: skin intrinsic fluorescence; FPG: fasting plasma glucose; 1hPG: 1-h plasma glucose; 2hPG: 2-h plasma glucose; HbA1C: glycated hemoglobin; Col: total cholesterol; HDL: high-density lipoprotein; TAG: triacylglycerol; LDL: low-density lipoprotein; INS: insulinemia; HOMA-IR: homeostatic model assessment for insulin resistance; m-ALB: microalbuminuria; HNE: hydroxynonenal; ADMA: asymmetrical dimethylarginine; ROS: reactive oxygen species

Wilcoxon Signed-Rank test; * Paired T-test

**Table 3:** Characteristics of NR group

	Baseline	HI 1-yr	N paired	p-value
DBP (mm Hg)	77.1 ± 10.8	79.2 ± 9.2	19	0.4982
SBP (mm Hg)	127.6 ± 15.7	136.8 ± 11.7	19	0.0429
Cardiac frequency (bpm)	68.9 ± 8.6	69.6 ± 7.1	18	0.5594
BMI (Kg/m^2^)	27.1 ± 3.5	27.1 ± 3.9	19	0.8933
Waist circumference (cm)	97.7 ± 9.4	96.7 ± 12.2	17	0.4736
Woman	93.6 ± 8.8	90.1 ± 13.3	7	0.2965
Man	100.6 ± 9.2	100.9 ± 9.8	10	0.9277
RHI (a.u.)	2.4 ± 0.7	2.4 ± 0.8	16	0.7715
AI (75 bpm%)	23.8 ± 20.2	20.7 ± 19.1	17	0.9573
HRV (a.u.)	37 ± 17.5	35.3 ± 16.2	17	0.7417
SIF (fluor. unit)	2.4 ± 0.6	2.2 ± 0.5	18	0.0459
FPG (mg/dL)	105.3 ± 16.7	106.1 ± 20.3	19	0.8142
1hPG (mg/dL)	214.3 ± 46.4	228.6 ± 58.2	19	0.0745
2hPG (mg/dL)	200.8 ± 77	221.4 ± 67.9	19	0.0916
HbA1C (%)	6.5 ± 0.6	6.3 ± 0.6	19	0.1114
HbA1C (mmol)	47.7 ± 6.7	45.8 ± 6.3	19	0.1059
Col (mg/dL)	202.8 ± 33.1	204.6 ± 42.4	19	0.8262
HDL (mg/dL)	52.4 ± 15.7	54.2 ± 16.6	19	0.3269
TAG (mg/dL)	117.6 ± 47.7	131.3 ± 71.2	19	0.2416
LDL (mg/dL)	126.9 ± 27.1	124.2 ± 30.8	19	0.6712
INS (mIU/L)	19.6 ± 23.4	13.5 ± 7.2	18	0.3750*
HOMA-IR	5.3 ± 6.8	3.7 ± 2.1	17	0.4586*
m-ALB (mg/dL)	38.9 ± 97.2	17.8 ± 32.8	17	0.0714*
HNE (ug/mL)	10.1 ± 7.7	6.8 ± 5.1	17	0.1634
ADMA (ng/mL)	67.4 ± 69.9	46.1 ± 20.4	17	0.2435*
ROS (umol/min)	0.20 ± 0.02	0.21 ± 0.02	14	0.8270

miR-21: circulating microRNA-21; DBP: diastolic blood pressure; SBP: systolic blood pressure; BMI: body mass index; RHI: reactive hyperemia index; AI: augmentation index; HRV: heart rate variability; SIF: skin intrinsic fluorescence; FPG: fasting plasma glucose; 1hPG: 1-h plasma glucose; 2hPG: 2-h plasma glucose; HbA1C: glycated hemoglobin; Col: total cholesterol; HDL: high-density lipoprotein; TAG: triacylglycerol; LDL: low density lipoprotein; INS: insulinemia; HOMA-IR: homeostatic model assessment for insulin resistance; m-ALB: microalbuminuria; HNE: hydroxynonenal; ROS: reactive oxygen species

T test.; * Wilcoxon test

The R subjects exhibited significantly reduced BMI, waist circumference (WC), RHI, 1hPG, HbA1c, INS, HOMA, m-ALB, HNE and ROS, but increased HDL (Table 2). No changes affected NR group (Table 3). When we detailed in R the changes from baseline to follow-up, accordingly with sex (Table 2), we noticed a reduction of BMI, RHI, insulinemia and HOMA in women, a weight loss more in women than men, which exhibited a reduction of WC, 2hPG and ROS, significantly. HbA1c and HNE levels are reduced for both, significantly; the changes in HNE are carried out more in men than in women.

### Dysglycemia predicts miR-21 responsiveness after HI-1 year

**Fig. 2:**
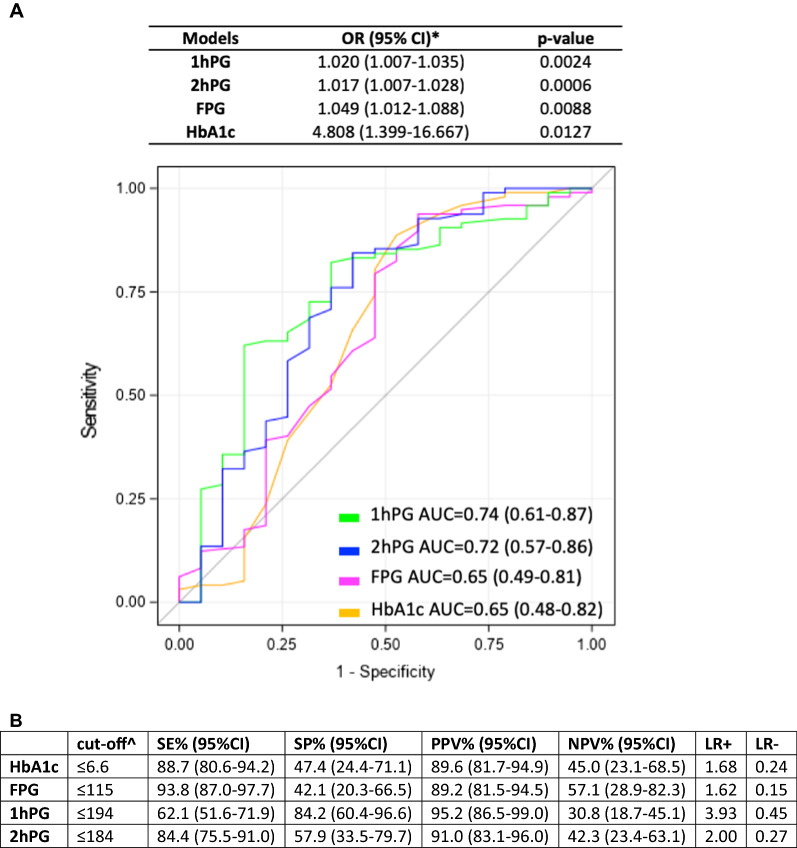
ROC curves of glycaemic parameters models in detecting miR-21 responders (R). **A** For each glycaemic parameter, a logistic model was implemented to evaluate the association with R. ROC curves were represented. Yellow-line represents ROC curve for HbA1c model, magenta-line represents ROC curve for FPG model, green-line represents ROC curve for 1hPG model and blue-line represents ROC curve for 2hPG model in discriminating between R and NR. Logistic regression models to evaluate the associations between lower miR-21 levels (at 1-yr) and glycemic parameters (baseline) were performed for predicting the effectiveness of the diet and relatives lower glycemic levels. **B** Best cut-offs of glycemic parameters in predicting miR-21 reduction were calculated using Youden index. Diagnostic values as sensitivity, specificity, positive and negative predicted values and positive and negative likelihood ratio were also evaluated

Logistic regression models and multiple ROC curves were drawn for the associations between glycemic parameters and the ability of the diet to reduce miR-21. We observed that for each unit-decrease in 1hPG and 2hPG we expected about 2% increased probability in discriminating Responder group (low-miR-21) after HI. In addition, for 1-unit decrease in HbA1c the probability in the discriminating Responder group is 4.8 times the probability of NR group. Best AUC was observed for 1hPG (0.74) (Fig. 2A), whereas best cut-offs of glycemic parameters in predicting miR-21 reduction and related diagnostic values (Fig. 2B).

In particular, the stratification of glycemic parameters (Table 4), categorized on ADA guidelines for prediabetes and diabetes, highlights that subjects with 2hPG ≥ 200 mg/dL have a minor probability to reduce miR-21 after HI (R group), versus 2hPG < 140 mg/dL or versus 2hPG 140–199 mg/dL. Lower values of FPG lead to a greater probability in discriminating R group if compared with higher values, although not significant (OR = 7.33; p = 0.0604). Moreover, values of HbA1c between 5.7 and 6.4% lead to almost 4-time greater probability in discriminating R group if compared with subjects with HbA1c ≥ 6.5 (p = 0.0128).

**Table 4:** Univariable logistic models for the association between Responders and baseline glycemic parameters

Model	OR (95% CI)	p-value
HbA1c		
≥ 6.5	1.00 (Ref.)	
5.7–6.4	3.89 (1.33–11.32)	0.0128
< 5.7	0.80 (0.13–5.08)	0.8130
FPG		
≥ 126	1.00 (Ref.)	
100–125	3.63 (0.44–29.92)	0.2316
< 100	7.33 (0.92–57.71)	0.0604
2hPG		
≥ 200	1.00 (Ref.)	
140–199	4.57 (1.38–15.11)	0.0102
< 140	6.00 (1.53–23.53)	0.0127

OR: Odds Ratio; CI: confidence interval; HbA1C: glycated hemoglobin; FPG: fasting plasma glucose; 2hPG: 2-h plasma glucose; Ref: reference category

At linear regression analysis (Additional file 1: Table S3), all glycemic parameters showed a significant association with miR-21 values, with parameter estimates ranging from 0.005 to 1.7. Moreover, ROS was highly associated with 1hPG and 2hPG: for 0.01 unit decrease in ROS, we expected a decrease of 12.7 and 16.3 unit of 1hPG and 2hPG, respectively.

## Discussion

Preventive programmes for the global containment of non-communicable diseases (NCD) are centred on the reversibility of dysglycemia, especially for diabetes and its cardiovascular burden, which the World Health Organization (WHO) and the Diabetes Prevention Program (DPP) estimated to be increasing.

In the present work, we sought to demonstrate whether epigenetic changes, based on miR-21 influence, may occur during a habit intervention, in which MedDiet structured by Harris & Benedict could be an eligible method to retain hyperglycemia.

Mediterranean diet (MedDiet)—largely based on plant foods and therefore includes a lot of fruits and vegetables, beans and pulses, nuts and seeds, whole grains and olive oil—notoriously demonstrated beneficial effects on intermediate markers of cardiovascular risk [36]. MedDiet can promote weight loss and improve blood glucose management in people with type 2 diabetes, in metabolic syndrome [37], on endothelial function in T2D [38, 39] and on its capacity for decreasing oxidative stress.

In this work, habit intervention (HI) was able to reduce oxidative stress and the relative axis miR-21/ROS/HNE, interconnected each other as demonstrated by the correlation matrix (Additional file 1: Table S1). In recent work, we demonstrated that the increased glycemic levels are strongly associated with the increased levels of circulating parameters of vascular damage induced by oxidative stress [28]. Furthermore, in our published study about miRs effects on oxidative stress, we showed the protective effects of miR-21 silencing in an experimental model of endothelial cells exposed to glucose variability (GV): functional inhibition of miR-21 lead to upregulation of antioxidant genes, inhibited by GV [27]. The functional over-expression of miR-21 determined the inhibition of ROS-homeostasis target genes, e.g. Krev/Rap1 interaction trapped-1 (KRIT1), Foxo1 and some genes related to antioxidant response, such as Nuclear Erythroid 2 related factor-2 (NRF2) and superoxide-dismutase-2 (SOD2), explaining the causal link between ROS formation and miR-21 [27].

For the first time, in the present work, we noticed that the subjects with a lower-miR-21 (R group) phenotype in HI, besides reducing glycemia significantly, showed lower ROS and HNE levels than baseline. In addition, lower-miR-21 (R group) phenotype in HI exhibits an ameliorating endothelial function expressed as reactive hyperemia index, measured by EndoPAT, reduced BMI and visceral fat, the latter measured as waist circumference. Thus, the HI composed preferentially by MedDiet scheme (50–55% carbohydrates, 15–20% proteins, 25–30% lipids) was able to influence crucial epigenetic mechanisms that in hyperglycemia perpetuate the damage, making the phenomenon reversible. Indeed, it is notorious that hyperglycemia has central trigger factors been recognized as high and prolonged oxidative stress within tissues [40, 41]. The role of miRs in nutritional therapies has been assessed making them novel markers for a successful diet at the molecular level in particular for the prevention of the risk to the development of cardiovascular disease and also for preventing T2D development in subjects with CVD [42].

Gender differences also have been found in our data. The metabolic changes observed in our work during HI concerning baseline can be included in a pathway of genetic influences. In lower-miR-21 (R group) phenotype, women showed a reduction of glycemic parameters, insulinemia and HOMA, and a higher weight loss than in men, which exhibited a reduction in waist circumference (Table 2), mostly attributed to the effects of sex hormones on metabolism [43]. Recent literature evidenced that BMI reflects the major influence of genetic variants than epigenetics: in T2D, polymorphisms on the gene FTO (fat mass and obesity-associated), a gene associated with the common forms of obesity [44], IR in T2D [45], modulating insulin activity and BMI [46], seems to be dependent on the diet [47]. FTO regulates the amount of fat deposition and affects T2D risk through its effect on BMI; indeed, people homozygous for a particular FTO allele weighed about 3 kg more and had a 1.6-fold greater rate of obesity than those who had not inherited this trait.

BMI is less accurate for assessing healthy weight in some groups of people because it does not distinguish between the proportion of weight due to fat or muscle. Waist circumference is a better estimate of visceral fat, become therefore a more accurate predictor of cardiovascular risk, type 2 diabetes in men and metabolic syndrome.

In this study, we revealed putative pathogenic mechanisms exploring an epigenetic approach miR-based to evaluate the effective changes that occurred in lifestyle, measuring plasmatic levels of miR-21 axis, that globally reflects the ROS damage index. Since dysglycemic conditions may remain undetected for many years while silently promoting disease progression and cardiovascular events, a parallel analysis of miRNA values may be helpful for the predictive power over time.

Circulating miR-21 levels are still under-characterized in the diabetic population due to different methods of standardization applied among the populations.

The MIR21 gene, located in TMEM49 gene on chromosome 17q23.2 [48], has been well characterized in many complications diabetes-related, such as diabetic retinopathy [49], kidney fibrosis [50], diabetic nephropathy [19], and insulin resistance (IR) [19]. cardiovascular disease [51], and atherosclerotic plaques [52]. Furthermore, the miR-21 exerts its deleterious effects also on adipose tissues of subjects with diabetes and obesity [53].

We aimed to develop a miRNA-based method as a predictor of worse outcomes in a high-risk population to develop diabetes. Our findings showed the ability of basal glycemic levels to predict miR-21 levels after 1-year of HI, allowing to select of people eligible for habit-intervention efficacy.

All these findings allow us to assert that controlling also plasmatic miR21 levels might ameliorate the deterioration of insulin-resistance that occurs within the pathophysiologic progression towards diabetes. As HbA1c predicts the risk of developing complications, we sought correlations between miR-21 and HbA1c; a positive correlation was found between these factors after HI, suggesting a link to long-term tissue damage. Highlighting these findings, we posit that miR-21 could be an early index of metabolic derangements.

In our results we show that ROS production, quantitatively measured by EPR, the only technique capable of returning ‘intrinsic’ quantitative information of free radical levels [34] that provides absolute quantification of ROS, was reduced after HI. As reported in our previous work, miR-21 could be an important modulator of ROS homeostasis and antioxidant pathways, and defective antioxidant responses are one of the major causes of cellular damage [24]. In diabetes, hyperglycaemia often inhibits the defensive machinery [28, 54] accompanied by increased lipid peroxidation and reduced endogenous antioxidant levels in diabetic patients compared to controls [55, 56].

We showed that miR-21 affects also the index of insulin resistance, which may affect the mitochondrial dysfunction observed in previous studies [27]. During insulin resistance (IR), glucose metabolism converges in mitochondria via insulin signalling causing diabetes progression [57]. In our work, the ameliorating of IR found in our patients after HI might be explained as an improving of mitochondrial biogenesis and functions.

Interestingly, in this work, we noticed reduced levels of lipid peroxidation adducts (HNE) in plasma after HI. The abrogation of the downstream effects of dysglycemia could be attributable to MedDiet because of the reach of antioxidants properties.

This study demonstrated a strong interaction between MedDiet and miR-21 damaging-axis on glycemia after 1-year follow-up. The significant occurrence of reduced glycemia in Responders group (lower-miR-21 phenotype) may be explained by a higher adherence to MedDiet, than the NR group.

Altogether, our data strongly suggest that miR-21 could be considered a predictor of ROS damage before the onset of diabetes, in virtue of its reduction after a habit intervention. These elements strongly argue in favour of using circulating miR-21 as a screening tool in preventive initiatives.

## Conclusions

In conclusion, this work demonstrated the ability of the lifestyle changes in ameliorating cardiometabolic traits as well as damaging factors such as miR-21/ROS/HNE axis, clearly demonstrating the huge potentiality to reverse dysglycemia. Accordingly, reduced levels of miR-21 are associated with a reduced abundance of ROS and reduced HNE.

Overall, these findings suggest that an epigenetic approach is a feasible strategy for early detection of metabolic abnormalities and predict worse outcomes using miR-21/ROS/HNE axis as a preventive tool.

## Strengths and limitations

The major strength is that the downregulation of miR-21 damage-axis is related to diet, to lower glycemia and weight loss, even if the size of the population is not wide enough.

The limitation of this study may be attributed to lack of adherence to diet (whom data are not annotated), with no predilection in the use of specific components and foods that reach in antioxidants and fibres.

## Supplementary Information

**Additional file 1: Table S1.** Correlation matrix for miR-21, ROS and HNE after 1 year of HI and other variables. **Table S2.** MiR-21 values between Responders (R) and Non-Responders (NR) at baseline and after 1 year of HI. **Table S3.** Univariable linear regression models for the association between ROS or miR-21 and each glycemic parameter at 1-year follow-up after HI.

## Data Availability

Not applicable.
