# Supplementary material for: Lower miR-21/ROS/HNE levels associate with lower glycemia after habit-intervention: DIAPASON study 1-year later
Source: Cardiovasc Diabetol. 2022 Mar 4;21:35. doi: 10.1186/s12933-022-01465-0 (PMC8895587; doi:10.1186/s12933-022-01465-0)
Supplement: Supplementary file 1 — Additional file 1: Table S1. Correlation matrix for miR-21, ROS and HNE after 1 year of HI and other variables. Table S2. MiR-21 values between Responders (R) and Non-Responders (NR) at baseline and after 1 year of HI. Table S3. Univariable linear regression models for the association between ROS or miR-21 and each glycemic parameter at 1-year follow-up after HI. [file 12933_2022_1465_MOESM1_ESM.docx]

**Supplementary Table S1. Correlation matrix for miR-21, ROS and HNE after 1 year of HI and other variables**

|  | **miR-21** | | | **ROS** | | | **HNE** | | |
| --- | --- | --- | --- | --- | --- | --- | --- | --- | --- |
|  | rho | *p* | N | rho | *p* | N | rho | *p* | N |
| Age | -0.0214 | 0.8106 | 128 | -0.0397 | 0.7886 | 48 | 0.0306 | 0.6837 | 180 |
| DBP (mm Hg) | 0.0719 | 0.4197 | 128 | -0.0540 | 0.7154 | 48 | -0.1556 | **0.0370** | 180 |
| SBP (mm Hg) | 0.1318 | 0.1381 | 128 | -0.1298 | 0.3793 | 48 | -0.1253 | 0.0937 | 180 |
| CF (bpm) | -0.0488 | 0.5844 | 128 | 0.0188 | 0.8989 | 48 | -0.0174 | 0.8169 | 180 |
| BMI (Kg/m^2^) | -0.0273 | 0.7603 | 127 | 0.0282 | 0.8490 | 48 | -0.0728 | 0.3326 | 179 |
| WC (cm) | -0.0030 | 0.9731 | 125 | -0.1777 | 0.2373 | 46 | -0.0895 | 0.2363 | 177 |
| RHI (a.u.) | 0.1195 | 0.1916 | 121 | 0.2161 | 0.1493 | 46 | 0.1261 | 0.0993 | 172 |
| AI (75bpm%) | -0.0244 | 0.7911 | 120 | 0.1428 | 0.3436 | 46 | 0.1147 | 0.1353 | 171 |
| HRV (a.u.) | -0.0161 | 0.8612 | 120 | 0.0799 | 0.5977 | 46 | -0.0383 | 0.6188 | 171 |
| SIF (fluor. unit) | 0.0744 | 0.4094 | 125 | -0.0087 | 0.9532 | 48 | -0.0623 | 0.4157 | 173 |
| FPG (mg/dL) | 0.2538 | **0.0039** | 128 | 0.1829 | 0.2134 | 48 | -0.1437 | 0.0543 | 180 |
| 1hPG (mg/dL) | 0.3224 | **0.0002** | 127 | 0.5494 | **<.0001** | 48 | -0.1321 | 0.0787 | 178 |
| 2hPG (mg/dL) | 0.6918 | **<.0001** | 128 | 0.5602 | **<.0001** | 48 | -0.1156 | 0.1222 | 180 |
| HbA1C (%) | 0.2652 | **0.0027** | 126 | 0.2832 | 0.0511 | 48 | -0.0147 | 0.8459 | 178 |
| HbA1C (mmol) | 0.2668 | **0.0023** | 128 | 0.2976 | **0.0400** | 48 | -0.0102 | 0.8922 | 180 |
| Col (mg/dL) | 0.0174 | 0.8451 | 128 | 0.2112 | 0.1495 | 48 | 0.0249 | 0.7405 | 180 |
| HDL (mg/dL) | 0.0173 | 0.8461 | 128 | 0.2724 | 0.0610 | 48 | 0.3468 | **<.0001** | 180 |
| TAG (mg/dL) | 0.0484 | 0.5876 | 128 | 0.1046 | 0.4794 | 48 | -0.2714 | **0.0002** | 180 |
| LDL (mg/dL) | -0.0029 | 0.9737 | 128 | 0.0667 | 0.6526 | 48 | -0.0594 | 0.4280 | 180 |
| INS (mIU/L) | 0.0380 | 0.6703 | 128 | -0.1261 | 0.3931 | 48 | -0.1343 | 0.0740 | 178 |
| HOMA-IR | 0.0809 | 0.3659 | 127 | -0.1049 | 0.4830 | 47 | -0.1237 | 0.1010 | 177 |
| m-ALB (mg/dL) | 0.0874 | 0.3445 | 119 | 0.2122 | 0.1569 | 46 | -0.1482 | 0.0546 | 169 |
| miR-21 (a.u) | - | - | - | 0.5192 | **0.0002** | 48 | 0.1928 | **0.0299** | 127 |
| HNE (ug/mL) | 0.1928 | **0.0299** | 127 | 0.1111 | 0.4523 | 48 | - | - | 180 |
| ADMA (ng/mL) | 0.0192 | 0.8371 | 117 | -0.2855 | 0.0517 | 47 | 0.0150 | 0.8733 | 116 |
| ROS (umol/min) | 0.5192 | **0.0002** | 48 | - | - | 48 | 0.1111 | 0.4523 | 48 |

HI: habit intervention; DBP: diastolic blood pressure; SBP: systolic blood pressure; CF: Cardiac frequency; BMI: body mass index; WC: waist circumference; RHI: reactive hyperemia index; AI: augmentation index; HRV: heart rate variability; SIF: skin intrinsic fluorescence; FPG: fasting plasma glucose; 1hPG: 1-hour plasma glucose; 2hPG: 2-hours plasma glucose; HbA1C: glycated hemoglobin; Col: total cholesterol; HDL: high-density lipoprotein; TAG: triacylglycerol; LDL: low density lipoprotein; INS: insulinemia; HOMA-IR: homeostatic model assessment for insulin resistance; m-ALB: microalbuminuria; miR-21: circulating microRNA-21; ADMA: asymmetrical dimethylarginine; ROS: reactive oxygen species; HNE: hydroxynonenal.

**Supplementary Table S2. MiR-21 values between Responders (R) and Non-Responders (NR) at baseline and after 1 year of HI**

|  | **Baseline** | **HI 1-yr** |
| --- | --- | --- |
| Responders miR-21 (a.u.±sd) | 0.0385±0.0412 | 0.0037±0.0053 |
| Non-Responders miR-21 (a.u.±sd) | 0.0148±0.0144 | 0.0485±0.034 |

miR-21: circulating microRNA-21; HI: habit intervention. ****p<0.0001, **p<0.01, 1-way ANOVA with Tukey’s post-hoc multiparameter comparisons.

**Supplementary Table S3. Univariable linear regression models for the association between ROS or miR-21 and each glycemic parameter at 1-year follow-up after HI**

| **Outcome** | **Independent Variable** | **Parameter estimate*** | **Standard Error** | **P-value** |
| --- | --- | --- | --- | --- |
| HbA1c | ROS | 0.06279 | 0.03135 | **0.0511** |
| FPG | ROS | 1.38504 | 1.09775 | 0.2134 |
| 1hPG | ROS | 12.69654 | 2.84701 | **<.0001** |
| 2hPG | ROS | 16.30372 | 3.55413 | **<.0001** |
| HbA1c | miR-21 | 0.00546 | 0.00178 | **0.0027** |
| FPG | miR-21 | 0.21314 | 0.07238 | **0.0039** |
| 1hPG | miR-21 | 1.02859 | 0.27010 | **0.0002** |
| 2hPG | miR-21 | 1.71986 | 0.15992 | **<.0001** |

HbA1C: glycated hemoglobin; FPG: fasting plasma glucose; 1hPG: 1-hour plasma glucose; 2hPG: 2-hour plasma glucose. * Parameter estimate for 0.01-unit increase of ROS and for 0.001-unit increase of miR-21.

**Cartoon of the main characteristics of the study**

**
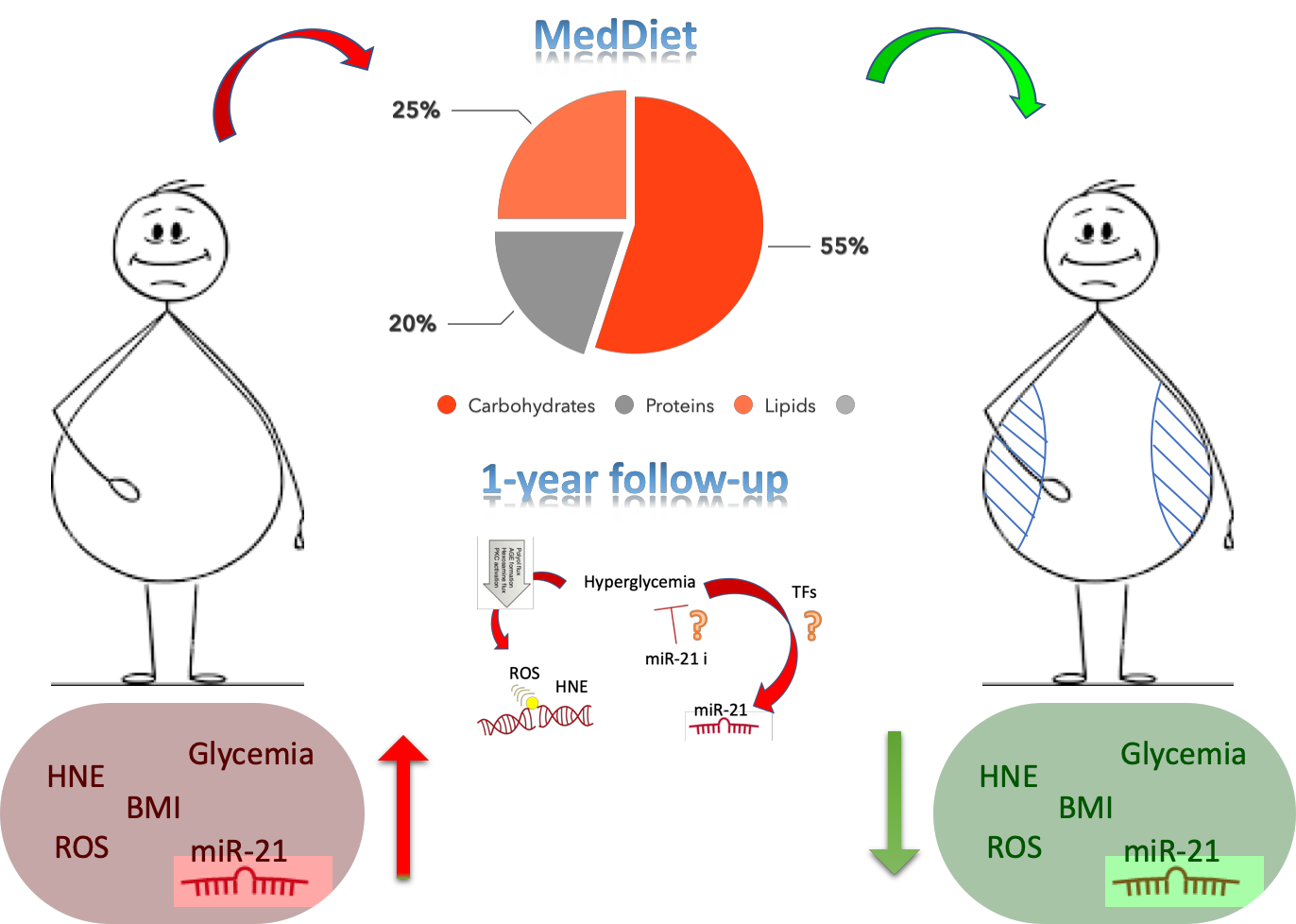
**

Depiction of major points of the study. The features of dysglycemic subjects are reported in this picture whose represents the phenotype of dysglycemic people unaware of their cardio-metabolic alterations. On the left, the population (N=531) before the habit-intervention (HI) showed higher levels of glycemia, BMI and circulating levels of miR-21/ROS/HNE axis than the same population after one-year of follow-up with the adoption of MedDiet, on the right. In the middle box, the mechanism proposed for the miR axis action. Hyperglycemia induces the increase of circulating miR-21 levels and of reactive oxygen species (ROS). It is well acknowledged the ROS induction by hyperglycemia as major component for the development of cardiovascular complications of diabetes. The reason why hyperglycemia determines the elevation of miR-21 would be ascribed to the regulation of transcriptional control by transcription factors (TFs) that bind to DNA in miR-21 transcription sites (ref 48). The direct effects of miR-21 inhibition on the mechanisms controlling the tissue glucose uptake is unknown.
